# FOSL2 Is Involved in the Regulation of Glycogen Content in Chicken Breast Muscle Tissue

**DOI:** 10.3389/fphys.2021.682441

**Published:** 2021-07-06

**Authors:** Xiaojing Liu, Lu Liu, Jie Wang, Huanxian Cui, Guiping Zhao, Jie Wen

**Affiliations:** ^1^State Key Laboratory of Animal Nutrition, Institute of Animal Sciences, Chinese Academy of Agricultural Sciences, Beijing, China; ^2^College of Animal Science and Technology, College of Veterinary Medicine of Zhejiang A&F University, Hangzhou, China

**Keywords:** chicken, muscle glycogen, FOSL2, CEBPB, gene network

## Abstract

The glycogen content in muscle of livestock and poultry animals affects the homeostasis of their body, growth performance, and meat quality after slaughter. FOS-like 2, AP-1 transcription factor subunit (FOSL2) was identified as a candidate gene related to muscle glycogen (MG) content in chicken in our previous study, but the role of FOSL2 in the regulation of MG content remains to be elucidated. Differential gene expression analysis and weighted gene coexpression network analysis (WGCNA) were performed on differentially expressed genes (DEGs) in breast muscle tissues from the high-MG-content (HMG) group and low-MG-content (LMG) group of Jingxing yellow chickens. Analysis of the 1,171 DEGs (LMG vs. HMG) identified, besides *FOSL2*, some additional genes related to MG metabolism pathway, namely *PRKAG3*, *CEBPB*, *FOXO1*, *AMPK*, and *PIK3CB*. Additionally, WGCNA revealed that *FOSL2*, *CEBPB*, *MAP3K14*, *SLC2A14*, *PPP2CA*, *SLC38A2*, *PPP2R5E*, and other genes related to the classical glycogen metabolism in the same coexpressed module are associated with MG content. Also, besides finding that *FOSL2* expression is negatively correlated with MG content, a possible interaction between *FOSL2* and *CEBPB* was predicted using the STRING (Search Tool for the Retrieval of Interacting Genes) database. Furthermore, we investigated the effects of lentiviral overexpression of FOSL2 on the regulation of the glycogen content *in vitro*, and the result indicated that FOSL2 decreases the glycogen content in DF1 cells. Collectively, our results confirm that FOSL2 has a key role in the regulation of the MG content in chicken. This finding is helpful to understand the mechanism of MG metabolism regulation in chicken and provides a new perspective for the production of high-quality broiler and the development of a comprehensive nutritional control strategy.

## Introduction

Muscle glycogen (MG), as the main source of glucose for muscle glycolysis (Godfrey and Quinlivan, 2016), can supply adenosine triphosphate to muscles through its own degradation, which is closely related to muscle growth and development (Puolanne and Immonen, 2014). Under special physiological conditions (such as intense exercise), it can maintain the stability of blood sugar and regulate the balance of glucose metabolism. In addition, MG content affects various meat quality indicators, such as meat color, tenderness, and pH of livestock and poultry meat (Henckel, 1996; Berri et al., 2007; Le Bihan-Duval et al., 2008). The degradation of MG to lactic acid changes the pH, which affects meat quality; thus, pH is an important indicator of meat quality (Alnahhas et al., 2015).

A previous genome-wide association study (GWAS) analysis of the MG phenotype conducted using the whole-genome resequencing data of 474 chickens identified FOS-like 2, AP-1 transcription factor subunit (*FOSL2*) transcription factor subunit as a candidate gene whose product affects MG content (Liu et al., 2020). *FOSL2* belongs to the activator protein 1 (AP1) transcription factor family, which is expressed in various tissues and is involved in fat metabolism, bone development, and the pathogenesis of various diseases, such as cancer (Foletta et al., 1994; Chinenov and Kerppola, 2001; Eferl and Wagner, 2003). A study on the involvement of FOSL2 in type 2 diabetes found that the epigenetic downregulation of FOSL2 mRNA and protein expression levels by DNA hypermethylation may induce the occurrence of type 2 diabetes in patients (Li et al., 2016). FOSL2 plays a key role not only in human physiology and disease (Eferl et al., 2008), but also in the differentiation of chondrocytes by controlling osteoclast-specific transcription (Karreth et al., 2004). Additionally, FOSL2 regulates bone formation by osteoblasts in mouse and human mainly by regulating the expression of collagen isoforms collagen, type I, alpha 2 chain (COL1A2) and bone gamma-carboxyglutamate protein (BGLAP) (Bozec et al., 2010). Previous studies have reported that the *FOSL2* gene protein product activates *CEBPB* transcription in prostaglandin E_2_ (PGE_2_)–activated osteoblasts. Although the regulatory effect of transcription factor CEBPB on chicken MG content has also been reported (Sibut et al., 2011), the role of *FOSL2* expression in tissues other that bone tissue, such as its role in MG metabolism, has not been described to date. An interesting experiment conducted in mice found that osteoblasts specifically express *FOSL2* to regulate Adipoq and Bglap levels, which in turn influences systemic glucose and insulin metabolism (Bozec et al., 2013). Although the study did not clearly reveal that FOSL2 affects the content of MG, it suggested that FOSL2 is closely related to glucose metabolism. MG is synthesized from monomers of uridine diphosphate glucose and serves as a storage form of energy in the body. Thus, MG and glucose are inseparably related.

In this study, breast muscle tissues from the high-MG-content (HMG) group and the low-MG-content (LMG) group of Jingxing yellow (JX-Y) chickens were subjected to transcriptome sequencing. Combined with the weighted gene coexpression network analysis (WGCNA), the protein products of the identified candidate genes were further analyzed to study the effect of gene transcription on chicken MG content from the perspective of gene expression and its relationship with phenotype. Considering the complex function of FOSL2 in chickens of different breeds and sex, we studied the effects of the *FOSL2* expression level in breast muscle of Beijing You (BJY) chicken. Furthermore, we also investigated the effects of *FOSL2* expression on the MG content in chicken fibroblast DF1 cells. This study found that FOSL2 reduces MG content likely by downregulating the activity of *CEBPB*.

## Materials and Methods

### Ethics Statement and Animals

All animals and experimental protocols used in this study were approved by the Beijing Institute of Animal Science, Chinese Academy of Agricultural Sciences (the scientific research department responsible for animal welfare issues) (no.: IAS2019-21).

In this study, the experimental samples were derived from 474 female 98-day-old JX-Y chickens, a synthetic breed by the Institute of Animal Science of Chinese Academy of Agricultural Sciences, with high fat content and unique flavor, which is a better material for meat quality research. Additionally, 30 male and 30 female 98-day old BJY chickens, a unique Chinese local breed, with high flavor characteristics, were selected to evaluate the influence of the breed and sex.

All experimental birds were raised in an environmentally controlled room, in three-story step cages. Basal diets were formulated based on the National Resource Council (1994) requirements and the Feeding Standards of Chickens established by the Ministry of Agriculture, Beijing, China (2004).x1 All birds were provided *ad libitum* access to feed and water up to 98 days of age. All chickens were individually euthanized using carbon dioxide anesthesia and exsanguination by severing the carotid artery at 98 days of age after 12-h fasting (no additional feed was supplied, and the feed trough was not emptied). After slaughtering, the skin was cut open, and internal tissue samples were collected along the direction of muscle fibers. The pectoralis major muscle samples were weighed, snap-frozen in liquid nitrogen, and stored at −80°C for determination of MG, RNA sequencing, and fluorescence quantitative real-time polymerase chain reaction (qRT-PCR).

### Measurement of MG Content

Muscle Glycogen Assay Kit from Nanjing JianCheng Bioengineering Institute (Nanjing, China) was selected. Tissue samples of approximately 100 mg were first cut, with a fluctuation of no more than 0.5 mg among samples. Then, the breast muscle tissue samples were hydrolyzed with 300 μL of lye in a boiling water bath for 20 min and cooled afterward with running water. Subsequently, 0.1 mL of a 5% MG detection solution and 0.9 mL distilled water were added to the hydrolyzed samples and boiled for 5 min. Then, 2 mL of color reagent was added, and the optical density (OD) value was measured at a wavelength of 620 nm using a spectrophotometer. Finally, the MG content of the tested sample was calculated.

### Samples Collection for RNA Sequencing and qRT-PCR Analysis

The MG content of the JX-Y chicken female population ranged from 0.2 to 9.0 mg/g (Supplementary Material 1). We included individuals with an MG content of less than 1 mg/g in the LMG group and individuals with MG content of more than 4 mg/g in the HMG group. Transcriptome sequencing was performed on samples from 16 chickens (including three individuals in the HMG group, three individuals in the LMG group, and 10 individuals with random phenotypes). A total of six chickens in the HMG and LMG groups were analyzed by differential gene expression analysis to identify the differentially expressed genes (DEGs) between the two groups. In order to exclude the differences in FOSL2 gene expression caused by extreme phenotypes, we included 10 individuals with random phenotypes on the basis of six individuals for WGCNA. The MG content of BJY chicken was measured in 30 male and 30 female individuals. The MG content in male BJY chickens ranged from 0.8 to 5.1 mg/g, and in female BJY chickens, it ranged from 0.8 to 4.2 mg/g (Supplementary Material 1). We considered male and female individuals with MG content of less than 1 mg/g as the LMG group, and individuals with MG content of more than 2 mg/g as the HMG group. Fluorescence qRT-PCR analysis was performed on six male individuals and six female individuals (three individuals in the HMG group and three individuals in the LMG group).

### RNA Extraction and Sequencing

Total RNA was extracted from the breast muscle tissue samples using TRIzol reagent (Invitrogen, Carlsbad, CA, United States). The quality of the RNA was assessed after separation by electrophoresis on a 1% agarose gel, and RNA concentration was determined by spectrophotometry using a NanoDrop 2000 spectrophotometer (Thermo Fisher Scientific Inc., Waltham, MA, United States). The OD 260/280 values of all samples were within the range of 1.8 to 2.0 for RNA sequencing and qRT-PCR analysis.

We used a cDNA library construction method previously described by Chen et al. (2019). The mRNA was enriched by binding of the mRNA poly-A tail to oligo (dT)–coated magnetic beads and fragmented into small pieces. Single- and double-stranded cDNAs were synthesized using mRNA as a template. The double-stranded cDNA was purified using the QIAQuick PCR purification kit (QIAGEN, Valencia, CA, United States). After purification, end repair, and ligation to sequencing adapters, agarose gel electrophoresis was used for fragment separation and fragment size selection. Finally, PCR enrichment was performed to obtain the final cDNA library. RNA sequencing was performed on an Illumina NovaSeq 6000 platform (Illumina Inc., San Diego, CA, United States) by Frasergen (Wuhan, China), and 150-bp paired-end reads were generated (Supplementary Material 2).

After quality control, the HISAT2 software (Kim et al., 2015) was used to align the second-generation sequence of each sample to the reference genome sequence galGal6.0. The number of reads compared to the transcript of the sample was counted by the comparison result of bowtie2 called by RSEM (Li and Dewey, 2011) and converted by the FPKM (fragments per kilobase of transcript per million bases) method (Trapnell et al., 2010).

For biological duplication, we used the DEseq2 software to analyze the significance of differences in gene and transcript expression (Love et al., 2014) and used the edgeR software for differential expression analysis without biological duplication. The standard for screening the total DEGs is | log2FC| > 1.

### qRT-PCR Analysis

All PCR primers were designed at or just outside exon/exon junctions to avoid the amplification of residual genomic DNA using the Primer-BLAST on the National Center for Biotechnology Information (Bethesda, MD, United States) website, and specificity was determined using BLASTN (Supplementary Material 3). First-strand cDNA was synthesized using 2 μg of total RNA, random primers, and oligo(dT) primers, with the Transcriptor First Strand cDNA synthesis Kit (Takara, Dalian, China) following the manufacturer instructions. Target mRNAs were quantified by qRT-PCR analysis using SYBR Green Master Mix (Takara). The qRT-PCR reaction of each sample was performed on the QuantStudio 7 Flex system (Applied Biosystems, Shanghai, China) using 40 cycles (95°C for 3 min, 95°C for 3 s, and 60°C for 34 s). The amplification reaction for each sample was performed in triplicate. The collected data were analyzed using the 2^–ΔΔ*CT*^ method (Livak and Schmittgen, 2001), and all the results were normalized to the β-actin rRNA gene (Livak and Schmittgen, 2001; Liu et al., 2019).

### WGCNA

Network analysis was performed using WGCNA R package version 1.66 (Langfelder and Horvath, 2008). The WGCNA methods have been successfully applied to gene expression data from microarrays (Kadarmideen et al., 2011) and RNA sequencing platforms in animal studies (Kogelman et al., 2014). Before performing WGCNA analysis, the genes obtained through transcriptome sequencing were filtered to remove genes whose average expression level was less than 1. Next, we used the WGCNA program package to construct a weighted gene coexpression network. We first calculated the Pearson correlation between paired genes and constructed a Pearson correlation matrix based on all paired genes. Subsequently, the optimal β value (the β value of JX-Y chicken population = 14) that makes the gene distribution conform to the scale-free network was selected, and the weighted adjacency matrix was constructed. Then, based on the correlation expression value, the adjacency relationship matrix was converted into a topological overlap matrix, and each topological overlap matrix was used as a hierarchical clustering analysis (Zhang and Horvath, 2005). Finally, based on the topological overlap matrix, we used the dissimilarity between genes to cluster the genes and used the dynamic shearing method to cut the tree into different gene modules (i.e., gene clusters with high topological overlap).

After constructing the modules, we calculated the module eigengene (ME) (Zhao et al., 2010), which is defined as the first principal component of the standardized expression profiles. Module–trait relationships (Kogelman et al., 2014), assessed by Pearson correlation between the ME and phenotypic values, were used to select potential modules related to phenotypes. In addition, to assess the relationship between genes and traits, Pearson correlation between gene expression and phenotypic values was calculated and used as gene significance (GS) (Liu et al., 2016), and the average of the absolute GS values within each module was used to determine the module GS (MS).

### Comprehensive Analysis of PPI Network

We used the Search Tool for the Retrieval of Interacting Genes (STRING) database^1^ to assess protein–protein interaction (PPI) data (Szklarczyk et al., 2015). In addition, in order to determine the relationship between FOSL2 and CEBPB, we used the STRING database and converted the results visually by using the Cytoscape software.

### DF1 Cell Culture, Transfection, and Glycogen Detection

The chicken fibroblast DF1 cells were obtained from the cell bank of the Chinese Academy of Sciences. The DF1 cells were cultured in Dulbecco modified Eagle medium supplemented with 10% fetal bovine serum (Gibco, Grand Island, NY, United States) and 1% penicillin-streptomycin (Gibco). The cell lines were cultured at 37°C in a humidified incubator with 5% CO_2_ (Li et al., 2020).

The FOSL2 (Gene ID: 421416) overexpressing lentivirus and empty vectors were constructed by Ubigene (Guangzhou, China) and denoted as YOE-LV001-FOSL2 and YOE-LV001-Ctrl, respectively. Cells were plated in six-well plates at a density of 1 × 10^6^ cells per well, and YOE-LV001-FOSL2 and YOE-LV001-Ctrl were transfected into DF1 cells by the Polybrene, a transfection-promoting reagent provided by Ubigene. The formula for virus dosage is *V* (μL) = 1,000 × MOI × N/T, where MOI is multiplicity of infection, *N* is number of cells, and *T* is lentiviral infectious titer.

The transfected cells were selected with 2 μg/mL puromycin for 2 days. Using an inverted microscope, the imaging system is Olympus BX41, and the images are taken at a 100× field of view. Then cells were collected and tested for glycogen content. The amount of glycogen in DF1 cells was quantified using a glycogen content assay kit (Solarbio, BC0340, China) according to the manufacturer’s instructions (Zhao et al., 2017). The cell experiment was repeated three times with samples in triplicate.

### Statistical Analyses

The significance of the differences between groups was tested by the Student *t* test using the SPSS software version 22.0 (IBM Corp., Armonk, NY, United States). Confidence limits were set at 95% and *p* < 0.05 (^∗^) or *p* < 0.01 (^∗∗^) was considered significant. Data are presented as the mean ± standard error of the mean (SEM).

The GLM (general linear model) procedure in the SAS 9.4 software was used to analyze the differential expression of target genes of the HMG and LMG groups in BJY chickens, and the model was as follows:

$$y_{ij}=u+P_{j}+G_{i}+e_{ij}$$

where *y*_ij_ is gene expression quantity, *u* is population mean gene expression, *P_j_* is sex effect, *G_i_* is MG group effect, and *e*_ij_ is random error.

## Results

### Comparison of MG Content in the HMG and LMG Chicken Groups

**FIGURE 1:**
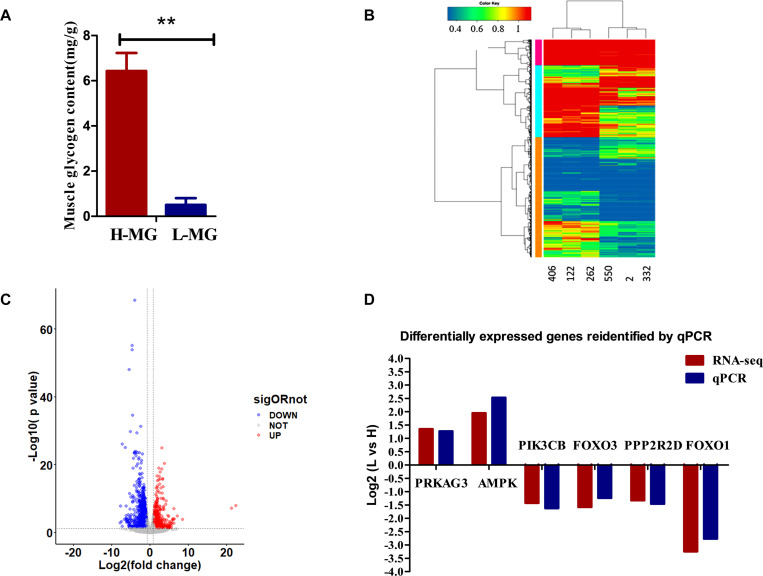
The phenotypic analysis and the RNA sequencing analysis results. **(A)** The MG content in chickens from the HMG and LMG groups (*P* = 0.0003). **(B)** Hierarchical clustering analysis. The hierarchical clustering analysis was performed based on DEGs; the heatmaps of all six samples revealed that the gene expression profiles in the same group were very similar. **(C)** Changes in gene expression profiles of Jingxing yellow (JX-Y) chicken from the HMG and LMG groups. Red dots represent upregulated genes, blue dots represent downregulated genes, and black dots (No) represent insignificantly DEGs. **(D)** Expression levels (fold change) of representative genes involved in MG metabolism pathways according to transcriptome analysis data of chickens from the HMG and LMG.

To study MG metabolism in breast muscle tissue from HMG and LMG groups of chickens, the MG content in breast muscle tissue samples was measured. The results revealed significant differences in the MG content between chickens from the HMG and LMG groups, as shown in Figure 1A. The MG content in the HMG group chickens was significantly (*p* < 0.01) higher than that in the LMG group chickens.

### Differential Expression Analysis of Gene Expression Data From HMG and LMG Chicken Samples

The hierarchical clustering analysis showed that only individuals within the same group clustered more closely (Figure 1B). The comparison of the results of the transcriptome sequencing analysis of individuals from the LMG group and HMG group identified a total of 1,171 DEGs, of which 376 were upregulated and 795 were downregulated (Figure 1C and Supplementary Material 4).

The expression of classic glucose metabolism genes in the HMG and LMG groups is shown in Figure 1D. In the HMG group, *AMPK* and *PRKAG* expression is higher than that in the LMG group (*p* < 0.01). The expression of other genes, such as *FOSL2* and *CEBPB*, was higher in the LMG group than that in the HMG group (*p* < 0.01).

### Construction of Weighted Gene Coexpression Network and Module Identification

A total of 15,934 genes were used to construct a weighted gene coexpression network. The gene modules shown in Supplementary Figure 1A were obtained based on the difference in hierarchical clustering. Based on the height value that quantifies the coexpressed similarity between modules, the modules with a value of less than 0.35 are merged. After merging, a total of 16 modules were identified. These coexpression modules are all represented in different colors in Supplementary Figure 1B, and the modules are named by the colors.

### *FOSL2* Modules Related to MG Traits

**FIGURE 2:**
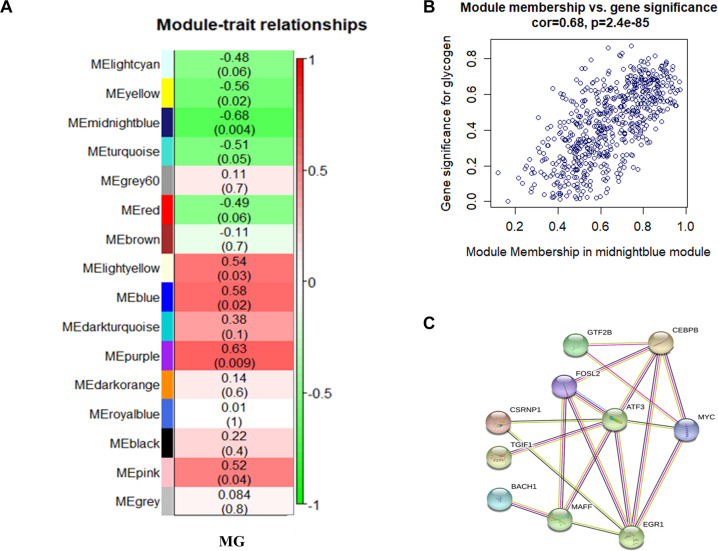
**(A)** Relationship between gene modules and MG traits in chicken breast muscle tissue. Each row in the table corresponds to a module, and each column to a trait. Each cell contains the corresponding correlation and *P* value. The module name is shown on the left side of the heatmap. The correlation is shown on the right side of the heatmap, in accordance with the color legend (red means positive correlation, and green means negative correlation). **(B)** Scatterplots of gene significance versus module membership for MG modules, along with the indicated correlation coefficients and *P* values. There is a highly significant correlation between GS and MM in the midnight blue module (cor. = 0.82, *P* = 5.9e-56). **(C)** Protein–protein interaction network. Each node represents a kind of protein, and the line between the nodes indicates that the pair of proteins has an interaction relationship.

We searched for the gene modules most related to MG traits by correlating 16 ME and MG traits (Figure 2A). The ME midnight blue module shows the most significant negative correlation with MG traits in breast muscle tissue (*r* = −0.68; *P* = 0.004), which suggests that the genes in this module may play a critical role in MG metabolism.

In the midnight blue module, 621 genes that are highly correlated with the corresponding modules and traits were clustered together (Figure 2B). Besides the candidate genes *FOSL2* and *CEBPB*, some genes related to the classical sugar metabolism pathway, such as *MAP3K14*, *SLC2A14*, *SLC12A4*, *PPP2CA*, *SLC38A2*, *PPP2R5E*, and *IGBP1*, were also found in the midnight blue module. This indicates that the protein products of the *FOSL2* and *CEBPB* genes and those of the sugar metabolism pathway-related genes may coparticipate in similar biological pathways and coregulate MG metabolism. The data in Supplementary Material 5 show the correlation between the aforementioned classic glucose metabolism–related genes and MG traits. The results reveal that the expression of the *FOSL2* and *CEBPB* genes is negatively correlated with MG content. The network analysis of all the genes of the midnight blue module in the JX-Y chicken WGCNA using the STRING software to predict the structure of the protein network revealed that there is an interaction between FOSL2 and CEBPB (Figure 2C).

### *In vivo* Validation of FOSL2 Gene Expression in Different Populations

In order to verify whether *FOSL2* and *CEBPB* can play the same role in the regulation of the MG content in different breeds and different sex groups, BJY chicken from the HMG and LMG groups were used for quantification of their expression.

The HMG and LMG groups included six chickens (three males and three females). The transcript abundance of *FOSL2* and *CEBPB* was verified by qRT-PCR analysis. The data in Table 1 show that the transcript abundance of the *FOSL2* and *CEBPB* was significantly upregulated in the LMG group (*p* < 0.01). These results were consistent with the results of the transcriptome analysis of JX-Y chicken muscle tissue, indicating that *FOSL2* and *CEBPB* also play a role in reducing MG content in BJY chicken.

**TABLE 1:** The relative quantification (RQ) of mRNA levels of *FOSL2* and *CEBPB* in BJY chicken HMG and LMG groups.

	Group	RQF	RQC	MG
MG	High (*n* = 6)	0.82	0.82	3.06
	Low (*n* = 6)	1.30	1.33	0.89
Gender	M (*n* = 6)	1.15	1.15	2.23
	F (*n* = 6)	0.96	0.99	1.71
*P* value	SEM	0.09	0.06	0.53
	MG	0.03	0.01	0.00
	Gender	0.31	0.30	0.25
	MG × gender	0.06	0.01	0.28

*M, male; F, female; RQF, relative quantification (RQ) of mRNA levels of FOSL2; RQC, relative quantification (RQ) of mRNA levels of CEBPB.*

### *In vitro* Validation of FOSL2 Gene

**FIGURE 3:**
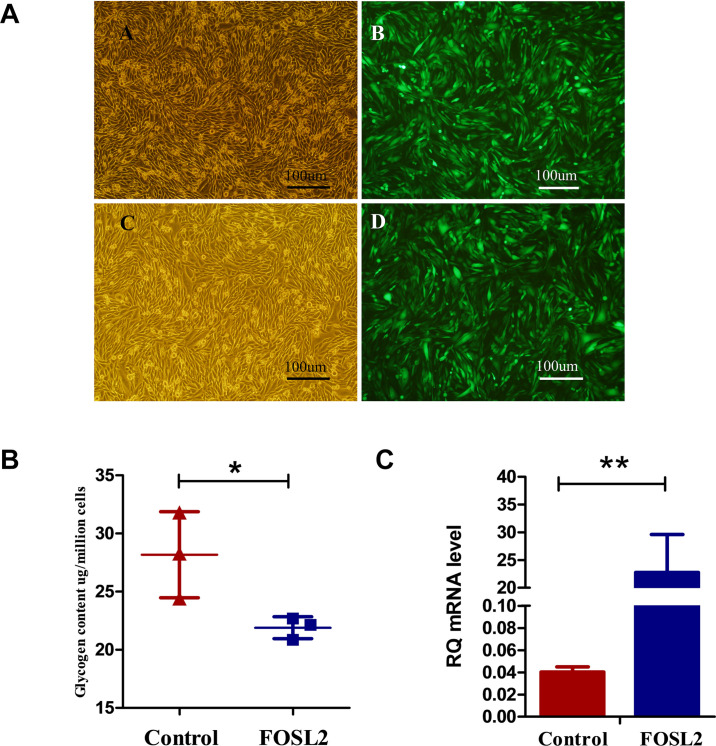
**(A,B)** Bright-field images and fluorescence microscopy images of transfected cells of chickens from the control group, respectively. Bright-field images and fluorescence microscopy images of cells from the FOSL2-transfected group, respectively. More than 80% of DF1 cells expressed green fluorescent protein. Scale bar = 100 μm. **(B)** The glycogen of control and FOSL2 overexpressing DF1 cells (*P* = 0.046). Control refers to the control group, and FOSL2 refers to the overexpression group of FOSL2. **(C)** Expression level of *FOSL2* gene by qRT-PCR in the control group and FOSL2 overexpression group (*P* < 0.001). Data are presented as the mean ± SEM. Control refers to the control group, and FOSL2 refers to the overexpression group of FOSL2.RQ: relative quantification.

We generated chicken fibroblast DF1 cells overexpressing *FOSL2* after lentiviral transfection, and the efficiency of *FOSL2* overexpression in DF1 cells was confirmed by fluorescence microscopy of transfected cells (Figures 3A,C). Enhanced green fluorescent protein (EGFP) is a mutant of green fluorescent protein (GFP), which emits more than six times of fluorescence intensity than GFP. EGFP plays a role only after gene expression. In addition, *FOSL2*-overexpressing DF1 cells had lower glycogen content compared with control group cells (Figure 3B). This is consistent with the results of our previous transcriptome analysis and WGCNA data analysis to mine FOSL2, which predicted their reduced MG content.

## Discussion

In the past few decades, livestock and poultry genetic breeding programs have focused on economically important traits, and significant progress has been made in genetic improvement. As an important intermediate in the process of sugar metabolism, MG interacts closely with blood sugar through glycogen synthesis. MG is not only involved in maintaining normal life activities of animals, but also affects the production performance and meat quality of livestock and poultry (Le Bihan-Duval et al., 2001; Berri et al., 2007; Alnahhas et al., 2014). However, the key genes that regulate the metabolism of MG in poultry have not yet been identified.

Numerous DEGs were identified in the HMG and LMG chicken groups. Among them, genes coding for proteins involved in the classical glycogen metabolism pathway, such as 5′-AMP–activated protein kinase (AMPK), which is a heterotrimeric protein that is involved in important signaling cascades to regulate cellular energy metabolism (Gowans and Hardie, 2014; Hardie and Ashford, 2014), Although this role of AMPK in glycogen metabolism has been widely established, increasing evidence suggests that the chronic activation of AMPK leads to glycogen accumulation instead of glycogen degradation (Hunter et al., 2011). For instance, it has been shown that the constitutive activation of AMPK via mutations in the γ2 and γ3 subunits leads to the accumulation of glycogen in the skeletal and cardiac muscles of pigs and mice (Milan et al., 2000; Ahmad et al., 2005; Luptak et al., 2007). According to the GeneCards^2^ database, the AMPK gamma subunit (*PRKAG3*), phosphatidylinositol-4,5-bisphosphate 3-kinase catalytic subunit beta (*PIK3CB*), protein phosphatase 2 regulatory subunit Bdelta (*PPP2R2D*), forkhead box O1 (*FOXO1*), and forkhead box O3 (*FOXO3*) are involved in the AMPK signaling pathway, thereby regulating MG content.

As expected, it was found that the expression of *FOSL2* and *CEBPB* genes in the breast muscle tissue of the HMG group is different from that of the LMG breast muscle tissue. The information in GeneCards (see text footnote 2) shows that FOSL2 activates CEBPB transcription in PGE_2_-activated osteoblasts. In addition, studies in the abdominal fat line broiler breeds bred by the French Ministry of Agriculture have shown that the downregulation of CEBPB affects MG production through a cAMP-dependent signaling pathway (Sibut et al., 2011). Although there is no relevant literature on the involvement of FOSL2 in the regulation of MG content through activation of CEBPB, the DEGs identified by transcriptome analysis of breast muscle tissue from HMG and LMG groups and the existing research results on MG indicate that FOSL2 may regulate the activation of CEBPB and thereby lead to changes in the MG content.

Some studies have reported that the traditional transcriptome sequencing statistical analysis methods have the drawback of not being able to distinguish confounding factors (Barabási and Oltvai, 2004; Ghaemi et al., 2019), but WGCNA can solve some of the analysis issues caused by confounding factors (Camacho et al., 2018; Emilsson et al., 2018; Mangul et al., 2019). Because of the aforementioned problems in transcriptome analysis, 10 individual chickens were randomly added on the basis of the original six individual chickens for transcriptome sequencing and WGCNA. In the WGCNA of JX-Y chickens, the *FOSL2* and *CEBPB* genes present in the ME midnight blue module are highly negatively correlated with MG traits. Besides the candidate genes *FOSL2* and *CEBPB*, this module also comprises many classic genes related to MG and sugar metabolism. For example, *MAP3K14*, *PPP2CA*, *SLC38A2*, *PPP2R5E*, *IGBP1*, etc. The above results indicate that the *FOSL2* and *CEBPB* genes are coexpressed and their protein products may have a coregulatory role. Furthermore, network analysis of the MG-related genes in the ME midnight blue module performed using the STRING software to predict the possible relationship between their proteins revealed a regulatory network between FOSL2 and CEBPB. Based on the above findings, we hypothesize that low expression of FOSL2 will inhibit the transcription of CEBPB, thereby decreasing MG production.

Differences in the selection process and selection intensity will lead to differences in traits of different breeds. We selected BJY chickens of different sex to quantitatively analyze the expression of the *FOSL2* and *CEBPB* genes. The results showed that FOSL2 and CEBPB are also differentially expressed in the HMG and LMG chicken groups. These results not only showed that sex did not affect the effect of FOSL2 and CEBPB on the reduction of MG content, but also that both proteins had an effect in reducing MG content in BJY chickens. Currently, there is no report on whether sex differences affect MG content in poultry, but studies in cattle suggest that sex differences do not affect MG content (Komatsu et al., 2015).

Members of the Fos gene family are attracting increasing attention as they may have multifunctional roles in a variety of physiological processes, including fat metabolism, bone development, and the pathogenesis of diseases, such as cancer. In this study, transcriptome analysis and WGCNA revealed that FOSL2 was related to MG content. Overexpression of FOSL2 in chicken fibroblast DF1 cells also confirmed its important role in decreasing the MG content.

At present, there are only a few studies on the key genes involved in MG content regulation in chicken, but more studies have been performed in pigs, cattle, and other animals. For instance, a major MG-related gene, namely, *PHKG1*, has been discovered in pigs (Ma et al., 2014). In the JX-Y chicken transcriptome analysis in this study, the *PHKG1* gene was not found to be differentially expressed. This may be due to the small sample size and differences between species. Although our study has certain limitations, it still advances the existing knowledge of chicken MG traits and provides a theoretical basis for subsequent research.

Conventional transcriptome sequencing analysis has been widely used in the study of various traits of poultry (Luo et al., 2019; Yang et al., 2020). In this study, we used basic differential expression analysis and gene coexpression network analysis to identify molecular network pathways that are involved in the regulation of glycogen content in chicken breast muscle. Furthermore, combined with the results of the whole-genome association analysis in the previous period, a key gene that affects MG content, namely, FOSL2, was identified. The results of the quantitative analysis of FOSL2 expression in BJY chickens showed that its regulatory effect on the MG content is not affected by breed or sex. The results of the analysis in DF1 cells further confirmed the negative regulatory effect of FOSL2 on MG content.

## Conclusion

Based on results from previous GWAS and WGCNA, we identified a key gene, namely, *FOSL2*, which negatively regulates MG content. Our data suggest that FOSL2 may decrease MG content by regulating its downstream gene *CEBPB*, as both genes exhibit the same pattern of expression. *In vivo* experiments showed that the effect of FOSL2 on MG was not affected by breed or sex. These results provide new insights and reference for the study of the regulation of MG metabolism.

## Data Availability Statement

The raw whole genome sequencing data reported in this paper have been deposited in the Genome Sequence Archive (Wang et al., 2017) in BIG  National Genomics Data Center Members and Partners (2020) under accession number CRA004003 that can be publicly accessed at https://bigd.big.ac.cn/gsa.

## Ethics Statement

All animals and experimental protocols used in this study were approved by the Beijing Institute of Animal Science, Chinese Academy of Agricultural Sciences (the scientific research department responsible for animal welfare issues) (No. IAS2019-21).

## Author Contributions

XL performed the study, analyzed the data, and drafted the manuscript. LL performed the study and drafted the manuscript. JWa analyzed the data and modified the manuscript. JWe, GZ, and HC contributed to the design of the study. All authors have read and agreed to the published version of the manuscript.

## Conflict of Interest

The authors declare that the research was conducted in the absence of any commercial or financial relationships that could be construed as a potential conflict of interest.

## Footnotes

1 https://www.string-db.org/

2 https://www.genecards.org/
