# Supplementary material for: FOSL2 Is Involved in the Regulation of Glycogen Content in Chicken Breast Muscle Tissue
Source: Front Physiol. 2021 Jul 6;12:682441. doi: 10.3389/fphys.2021.682441 (PMC8290175; doi:10.3389/fphys.2021.682441)
Supplement: Supplementary file 1 [file Image_1.pdf]

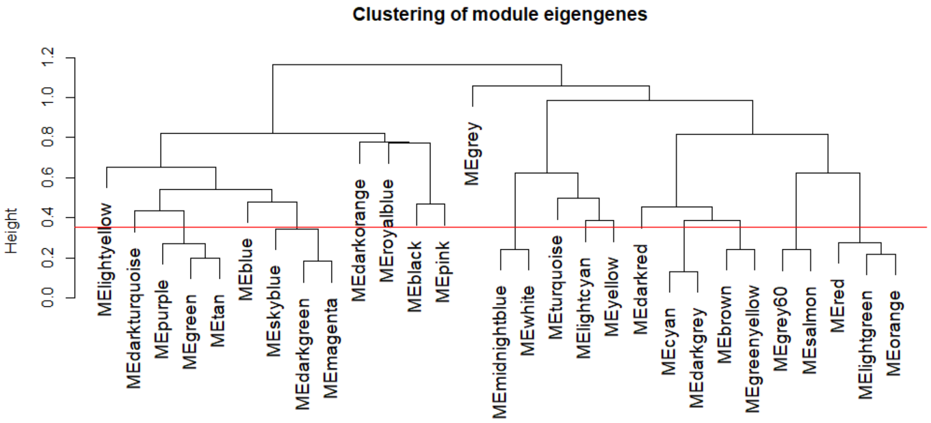

Figure S1a. Dendrogram of module eigengenes. Branches of the dendrogram group together module eigengenes that are positively correlated. Modules with height value less than 0.25 will be merged in subsequent analyses. The red line means height value is 0.35.

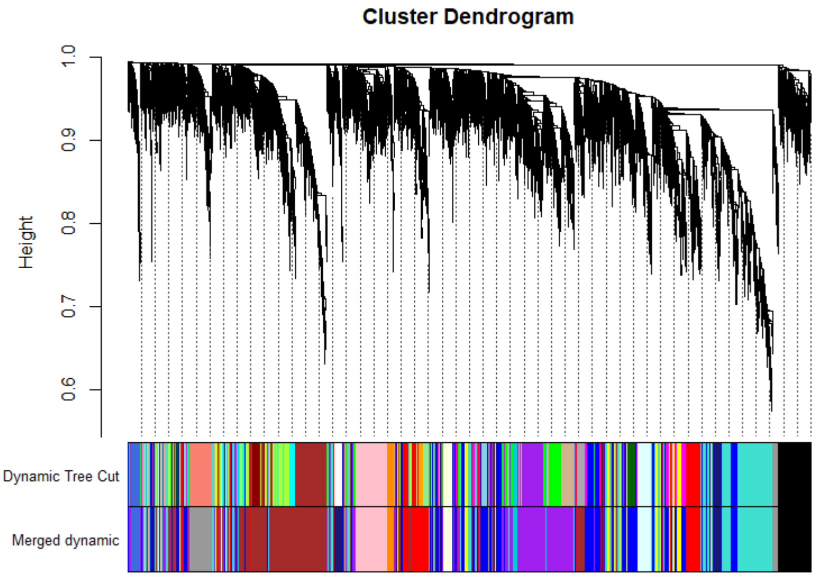

Figure S1b. Clustering dendrogram of gene profiles. Each short vertical line represents a gene and the branches are expression modules of highly interconnected groups of genes. The row under the dendrogram means the assigned original module and each color shows a gene module.
